# Analysis of fibrin networks using topological data analysis – a feasibility study

**DOI:** 10.1038/s41598-024-63935-7

**Published:** 2024-06-07

**Authors:** Martin Berger, Tobias Hell, Anna Tobiasch, Judith Martini, Andrea Lindner, Helmuth Tauber, Mirjam Bachler, Martin Hermann

**Affiliations:** 1 Data Lab Hell, Europastraße 2a, Zirl, Austria; 2 organLife Laboratory, Department of Visceral, Transplant and Thoracic Surgery, Medical University Innsbruck, Innsbruck, Austria; 3 Department of Anaesthesia and Intensive Care Medicine, Medical University of Innsbruck, Innsbruck, Austria; 4 Department of Urology and Andrology, District Hospital Hall, Hall in Tirol, Austria; 5 Department of Anaesthesiology and Intensive Care Medicine, Sanatorium Kettenbruecke der Barmherzigen Schwestern GmbH, Innsbruck, Austria; 6 Institute for Sports Medicine, Alpine Medicine and Health Tourism, UMIT - University for Health Sciences, Medical Informatics and Technology, Austria, Hall in Tirol, Austria

**Keywords:** Computational models, Bioinformatics

## Abstract

Blood clot formation, a crucial process in hemostasis and thrombosis, has garnered substantial attention for its implications in various medical conditions. Microscopic examination of blood clots provides vital insights into their composition and structure, aiding in the understanding of clot pathophysiology and the development of targeted therapeutic strategies. This study explores the use of topological data analysis (TDA) to assess plasma clot characteristics microscopically, focusing on the identification of the elements components, holes and Wasserstein distances. This approach should enable researchers to objectively classify fibrin networks based on their topologic architecture. We tested this mathematical characterization approach on plasma clots formed in static conditions from porcine and human citrated plasma samples, where the effect of dilution and direct thrombin inhibition was explored. Confocal microscopy images showing fluorescence labeled fibrin networks were analyzed. Both treatments resulted in visual differences in plasma clot architecture, which could be quantified using TDA. Significant differences between baseline and diluted samples, as well as blood anticoagulated with argatroban, were detected mathematically. Therefore, TDA could be indicative of clots with compromised stability, providing a valuable tool for thrombosis risk assessment. In conclusion, microscopic examination of plasma clots, coupled with Topological Data Analysis, offers a promising avenue for comprehensive characterization of clot microstructure. This method could contribute to a deeper understanding of clot pathophysiology and thereby refine our ability to assess clot characteristics.

## Introduction

The process of coagulation and fibrin polymerisation is a highly dynamic process, influenced by numerous factors determining the overall efficiency of clot formation^1^. Traditional coagulation tests such as prothrombin time (PT), activated partial thromboplastin time (aPTT) or fibrinogen measurements by Clauss method are part of the routine tests for coagulation and are used to detect derangements in this complex system. However, these tests describe mainly the velocity of the coagulation system until a detectable clot has been formed and are therefore primarily useful to assess the individuals risk for bleedings^2^. Recent advances in hemostasis research pointed out that there are substantial differences in further aspects of clot formation, such as the propagation velocity, the magnitude of the formed clot, or the retraction of fibrin fibres after initial formation, as well as the clot’s susceptibility to lysis^3,4^. Specific physical properties of the single fibrin fibers, such as fiber thickness or stiffness affect functional clot characteristics^5–7^. Thin fibers were found to have denser connected protofibrils than microscopically thicker appearing fibers, which also showed different resistance to lysis^5^ or bleeding risk and frequencies in patients with hemophilia, where initial concentrations of thrombin seem to alter not only clotting velocities but also three-dimensional clot formation characteristics^8,9^.

A clinical situation with acutely altered fibrin formation is dilution coagulopathy, which can arise after extensive fluid resuscitation with crystalloids or colloids to treat for example high blood loss in trauma patients^10^. The resulting lower clot stability and higher susceptibility to fibrinolysis potentially implicate clinical consequences such as time prolongation until hemostasis is reached in the acutely bleeding patient^11,12^. In addition, the fluids composition used for volume resuscitation was shown to differently affect the performance of the coagulation system, an observation that is thought to be connected to an altered clot architecture^13,14^.

Gaining a deeper insight into changes of fibrin networks is of substantial interest, as a precise characterization and quantification of the clot microstructure may shed light on important functional aspects of hemostasis. We here propose topological data analysis (TDA) as a novel mathematical approach to objectively quantify qualitative differences in fibrin networks imaged with confocal microscopy.

Topological data analysis is an analytical framework to identify the geometric structure within a dataset. TDA combines methods from algebraic topology as well as statistical analysis, which allows to measure the persistence of certain topological features and eventually to classify the data topologically. Images are analyzed by creating geometric structures like vertices, edges and surfaces out of pixels depending on their grayscale value. This enables to observe quantifiable items such as components and holes in an image. Applying this approach to fibrin networks, the qualitative differences of clot formation, theoretically, should exhibit alterations due to specific changes of the individual components of the coagulation system. Decreased concentration of coagulation zymogens by diluting the sample, or limiting thrombin functional capacity with an inhibitor could result in a specific pattern of structural changes. We propose that this pattern can be evaluated and compared by TDA.

## Materials and methods

The data analysis protocol was established using porcine blood samples and its applicability on human blood was investigated subsequently. TDA analysis was applied to plasma clots in static conditions. All experiments were conducted in accordance with the relevant guidelines and regulations.

### Porcine blood samples

Dilution of porcine plasma was performed in-vitro using frozen citrated plasma samples, stored at − 80 degrees Celsius. Those samples were collected according to national standards and with approval from the institutional ethics review board within the study with the protocol number BMWF-66.011/0080-II/3b/2011. Reporting is in accordance with current ARRIVE guidelines^15^. All experiments were performed at the Department of Anaesthesia and Intensive Care Medicine, Medical University Innsbruck. Venous blood samples from eight animals of either sex (healthy, adult animals; breed: domestic pigs “Deutsches Hausschwein”) were centrifuged at 3000 g for 10 min. Supernatants were collected to create a plasma pool of 18 mL. This amount was then divided into three portions of 6 mL, one of which was left untreated and further referred to as the baseline sample. The remaining two samples were diluted with either 4% gelatin (Gelofusin^®^, B. Braun Co., Melsungen, Germany) or 6% low molecular weight hydroxyethyl starch (HES, Voluven^®^, Fresenius Kabi AG, Germany). Dilution was carried out by adding 1.8 mL of each substance into a prepared 6 mL vial of pooled plasma.

### Human samples

This in-vitro analysis was performed with blood samples from healthy volunteers. The study was conducted according to the ethical principles defined by the Declaration of Helsinki, the study protocol was approved by the institutional review board of the Medical University of Innsbruck (EK number: 1215/2017, EK number: 1340/2021). Informed consent was provided by all volunteers prior any study related procedures were undertaken. Healthy subjects with an age range between 18 to 85 years were included if none of the following exclusion criteria were met: recent intake of medications that could interfere with the results of the study (such as anticoagulants or anti-platelet medications, nonsteroidal anti-inflammatory drugs such as ibuprofen or acetylsalicylic acid, selective serotonin reuptake inhibitors), pregnant or breast-feeding women as well as presence of hereditary or acquired coagulation disorders.

Citrated whole blood samples were spiked with different concentrations of argatroban (Argatra^®^, Mitsubishi Tanabe Pharma GmbH) ex-vivo. The concentration of the spiked argatroban levels corresponded to drug levels observed in vivo, 0.50 µg/mL (spiking step 1; S1) and 1.00 µg/mL (spiking step 2; S2). A baseline sample remained untreated except for the addition of 100 µL PBS (Phosphate buffered saline) to account for a dilution effect by adding the medication. After addition of the different doses of argatroban or PBS, whole blood samples were incubated for five minutes at room temperature on a roller mixer. Blood samples were centrifuged at 2500 g for 15 min to obtain plasma for coagulation analysis and confocal microscopy.

Global parameters of coagulation included aPTT (Siemens Pathromtin™ SL), PT (Siemens Thromborel S), and fibrinogen by Clauss method (Siemens Multifibren U), all measured on a BCS XP System (Siemens Healthineers) in the central laboratory of the Medical University Innsbruck. Due to the limited applicability of aPTT to porcine plasma these results are only presented for human samples^16^.

Results of global coagulation parameters were analyzed with GraphPad Prism version 10.1.0 using repeated measures one-way ANOVA (GraphPad Software, La Jolla, California USA, www.graphpad.com).

### Viscoelastic testing

Clot firmness was analyzed using the viscoelastic testing device ROTEM^®^ gamma (Tem Innovations, Instrumentation Laboratory Werfen), a point-of-care device to measure whole blood coagulation. Coagulation is induced by addition of calcium chloride (STAR-TEM, Tem Innovations, Instrumentation Laboratory Werfen) and tissue factor, which triggers the rapid activation of clot formation and polymerization of fibrin via the extrinsic pathway (recombinant tissue factor; EXTEM, Tem Innovations, Instrumentation Laboratory Werfen). The clotting time (CT) defines the elapsed time to activation of coagulation; clot formation time (CFT) represents the time between the beginning of clot activation and clot strength of 20 mm. Increasing strengthening through polymerization up to maximal clot formation is assessed by the maximal clot firmness (MCF). Normal values for results are 35–80 mm CT, 35 to 160 s CFT and 53 to 72 mm MCF. The amount of both baseline and diluted samples for measurement was 300 µL and temperature was steady at 37 degrees Celsius.

Raw images were exported from ROTEM devices and processed with Inkscape (version 1.0 beta1, www.inkscape.org).

### Confocal microscopy on forming clots

Real time live confocal visualization of fibrin networks was performed under static conditions in Lab-Tek 8-wellchambered #1.0 borosilicate cover glass slides (Nunc, Rochester, NY, USA). For this purpose 2 μL of fluorescent fibrin binding peptide (FFBP)^17^ were added to 200 µL of nondiluted/diluted citrated plasma into each well. Coagulation was induced via addition of 5 μL CaCl_2_ (0.2 mol/L CaCl_2_ in HEPES buffer, pH = 7.4) and 5 μL EXTEM (Tem Innovations, Instrumentation Laboratory Werfen). Real time live confocal microscopy was performed with a spinning disk confocal system (UltraVIEW VoX; Perkin Elmer, Waltham, MA, USA) connected to a Zeiss Axio Observer Z1 microscope (Zeiss, Oberkochen, Germany). Images and Z-stacks with a height of 10 µm were acquired using Volocity software (Perkin Elmer) using a 63 × oil immersion objective with a numerical aperture of 1.42.

### Image pre-processing

**Figure 1:**
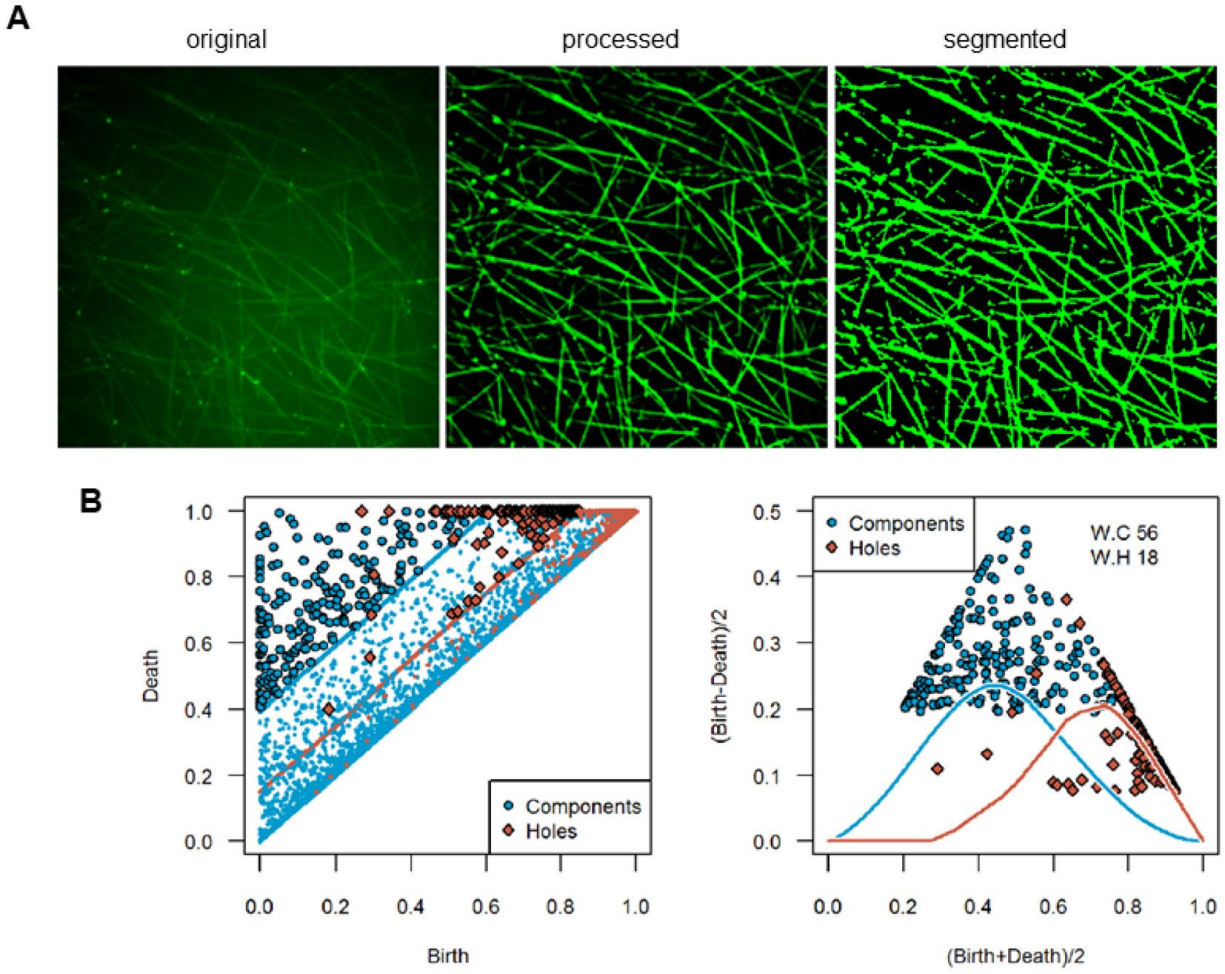
The (**A**) original (left), processed (center) and segmented (right) image of a human fibrin network after spiking with 0.5 µg/mL of argatroban (see also Fig. 3). (**B**) Persistence diagram to illustrate the mathematical processing using the segmented image. Blue points represent components, orange points holes.

The 3D greyscale images were averaged over the z-coordinate resulting in 2D greyscale images with a resolution of 1000 times 1000 pixels used for further analyses. To enhance image quality, image processing was conducted in MATLAB (Version R2017a) by applying box filtering and order-statistic filtering for noise reduction followed by histogram equalization to improve contrast and guided filtering to perform edge-preserving smoothing. Figure 1A shows an image before (original) and after image preprocessing (processed), resulting in the final dichotomized image (segmented).

### Topological data analysis

To quantify structural differences from baseline, topological data analysis (TDA) was applied to the 2D greyscale images to assess the change in topological features. For this purpose, we computed the features following the methodology presented in Wagner, Chen, and Vuçini 2012^18^ by using the C +  + software package DIPHA^19^. A cubical complex associated to each greyscale value from zero to one is defined as a set of vertices, edges and squares, generated by the pixels of the given data with lower or equally grey values. The higher the greyscale value, the more pixels are added to a complex. Then for every greyscale value the topology, i.e. holes and components, is computed. This is called the *persistence homology*.

Since pixels are subsequently added to the complex, topological features vanish, namely components merge and holes get filled, after considering enough pixels. This allows measuring the persistence of the topological features. Features with high persistence are considered more distinct than features with low persistence. This can be visualized with a *birth–death diagram*, also called *persistence diagram*, see Fig. 1B.

Every point in the persistence diagram corresponds to a topological feature computed from the greyscale image. Since different features can arise and vanish at the same greyscale values, a single point may represent multiple components or holes. The $x$-coordinate corresponds the grey value for which the feature was born (appeared), while the $y$-coordinate shows the value for which it died (vanished). Hence the persistence of the feature is given by the difference $y-x$. This implies that every point lies above the diagonal. Points with a large distance from the diagonal correspond to more distinct features. In Fig. 1B, blue points represent components and orange points holes. Due to the high resolution of the given data, a vast share of the computed features is due to noise. Hence, a threshold, i.e. a band around the diagonal, is computed in order to distinguish between distinct features and noise.

#### Computing thresholds

To compute thresholds in the persistence diagram which separate distinct features from features due to noise, all images were segmented in MATLAB (Version R2017a) using the *imbinarize* function. The number of features, i.e. components and holes, were automatically and precisely counted using *regionprops*. For all either porcine or human fibrin networks, the threshold for components and holes was separately set as the average distance in the persistence diagram from the diagonal matching the number of features counted in the segmented images (Fig. 1A). In addition, we provide the number of components and holes for each segmented image.

Points below the thresholds are plotted smaller and more transparent than points with high persistence. Note that introducing such a threshold reduces the number of topological features drastically (Fig. 1B).

#### Measuring differences

The Wasserstein distance is a commonly used metric to compare persistence diagrams^20^. Based on the calculated persistence homology, we calculate the Wasserstein distance using the TDA package in R to assess the change in the topological features^19,21,22^. We compute the Wasserstein distance from the persistence diagram consisting of the diagonal only as well as the Wasserstein distances from baseline persistence diagrams to the ones obtained either after dilution or spiking with argatroban.

The definition of Wasserstein distance is as follows: let *p* ≥ 1 then *p*th Wasserstein distance of two persistence diagrams *d*1 and *d*2 is defined as,

$$W_{p} \left( {d_{1} ,\,d_{2} } \right) = \left( {\inf\limits_{\sigma } {\sum\limits_{{x \in d_{1} }} {\left\| {x - \left. {\sigma \left( x \right)} \right\|_{\infty }^{p} } \right.} } } \right)^{\frac{1}{p}}$$

where σ ranges over all bijections from *d*1 to *d*2 and ||·||_∞_ denotes the infinity norm.

Remark. Since we added the diagonal to a persistence diagram the set of all bijections from *d*1 to *d*2 is non-empty.

For *n* ∈ N define a persistence diagram *d*_n_ = {(0, 2^−k^) | k = 1, . . . , n} ∪ ∆. Then it holds,

$$W_{p} \left( {d_{n} ,\,d_{n + k} } \right) \le \frac{1}{{2^{n} }},$$

and thus (*d*_n_)n ∈ N is a Cauchy sequence. But since the number of points above the diagonal goes to infinity as n → ∞, the limit is not a persistence diagram anymore. One therefore adapts the definition of persistence diagrams.

#### Comparing to fractal dimension

In addition, we compute the fractal dimension for the binarized images as the Hausdorff (Box-Counting) dimension to set our methodology into context with progressive dilution of human whole blood with isotonic saline presented in Ref.^23^.

The fractal dimension is given as,

$$\mathop {\lim }\limits_{\varepsilon \to 0} \frac{N\left( \varepsilon \right)}{{\log \left( {1/\varepsilon } \right)}}$$

where N( ∈) denotes the number of boxes of size ∈ needed to cover the non-zero pixels in the binarized images.

#### Weighted Silhouettes

Another method for analyzing persistence diagrams is weighted silhouettes given in Ref.^24^. Weighted silhouettes are functions defined as a weighted average over triangle functions, where each triangle function takes its maximum at a point of the persistence diagram. In Fig. 1B two silhouettes, one for components and one for holes, are visualized in the right diagram.

#### Assessing differences

In the case of spiking with argatroban, we assess the mean difference from baseline with respect to the Wasserstein distance from the diagonal by applying a paired t-test, with a significance level of 5%. In addition, we provide mean Wasserstein distances with corresponding 95% CIs. In the same way, we assess differences in fractal dimension, number of components and holes as well as several laboratory parameters.

A detailed description of applied mathematical definitions and formulae as well as further analyses of porcine samples are provided in the thesis of the first author^25^.

## Results

### Effect of thrombin inhibition or dilution on routine coagulation tests

To assess and verify the effect of the interventions, i.e. addition of the thrombin inhibitor argatroban and dilution of porcine plasma samples, global parameters of coagulation as well as viscoelastic tests were performed. Median baseline aPTT was 32 s (IQR 30–32), which increased for approximately 25 s after addition of 0.5 µg/mL (S1, 58 s, IQR 56–61) and 1.0 µg/mL argatroban (S2, 82 s, IQR 74–85, p = 0.0106). The determination of fibrinogen levels was similarly affected, returning decreased concentrations after each spiking step. The median baseline level of fibrinogen was 258 mg/dL (IQR 245–270), 230 mg/dL (IQR 224–237) after S1 and 185 mg/dL (IQR 184–192) after S2 (p = 0.0453).

**Figure 2:**
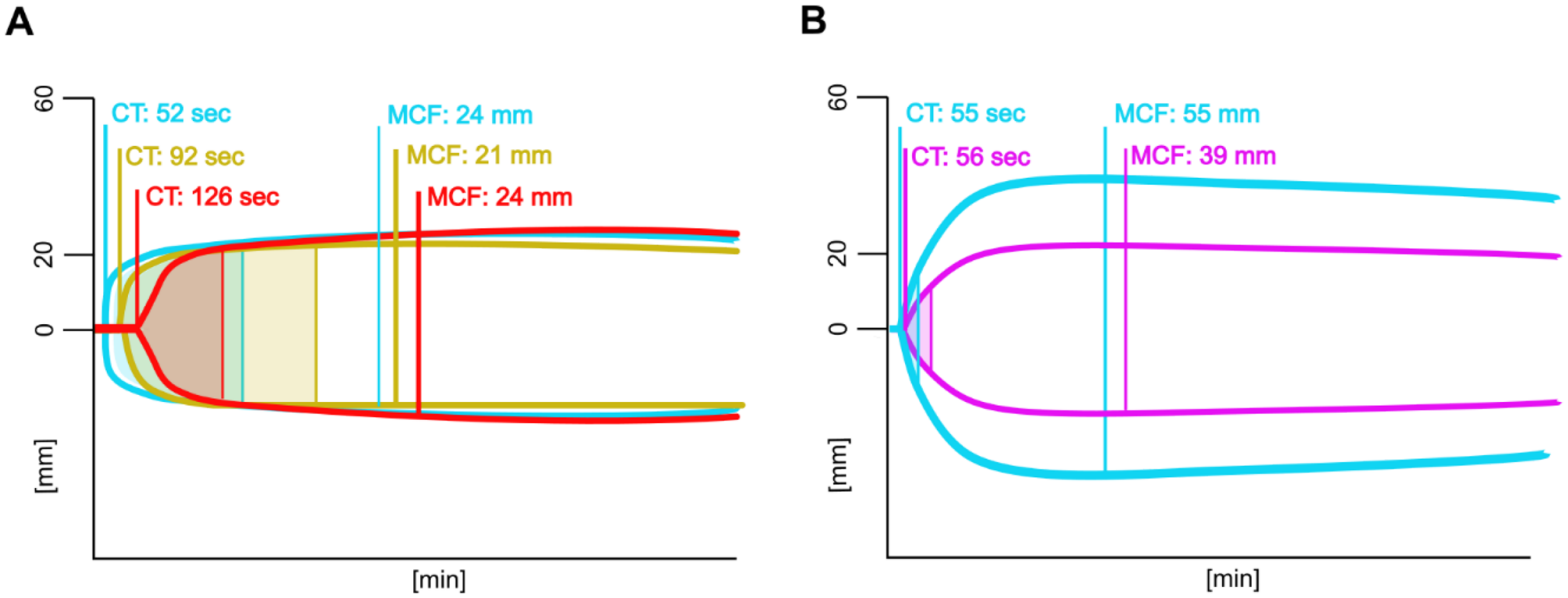
Viscoelastic measurements of (**A**) human plasma (blue) spiked with 0.5 µg/mL (yellow) or 1.0 µg/mL argatroban (red) and (**B**) porcine plasma (blue) diluted with a gelatin-based colloid (pink). *CT* clotting time, *MCF* mean clot firmness; Results of pooled samples (n = 3).

Viscoelastic tests similarly resulted in an increased time until clotting occurred, while the maximum clot firmness (MCF) did not differ between the treatments (Fig. 2A). Dilution of porcine plasma in contrast resulted in a lower MCF, while clotting times were only marginally affected (Fig. 2B).

### Structural changes after dilution

**Figure 3:**
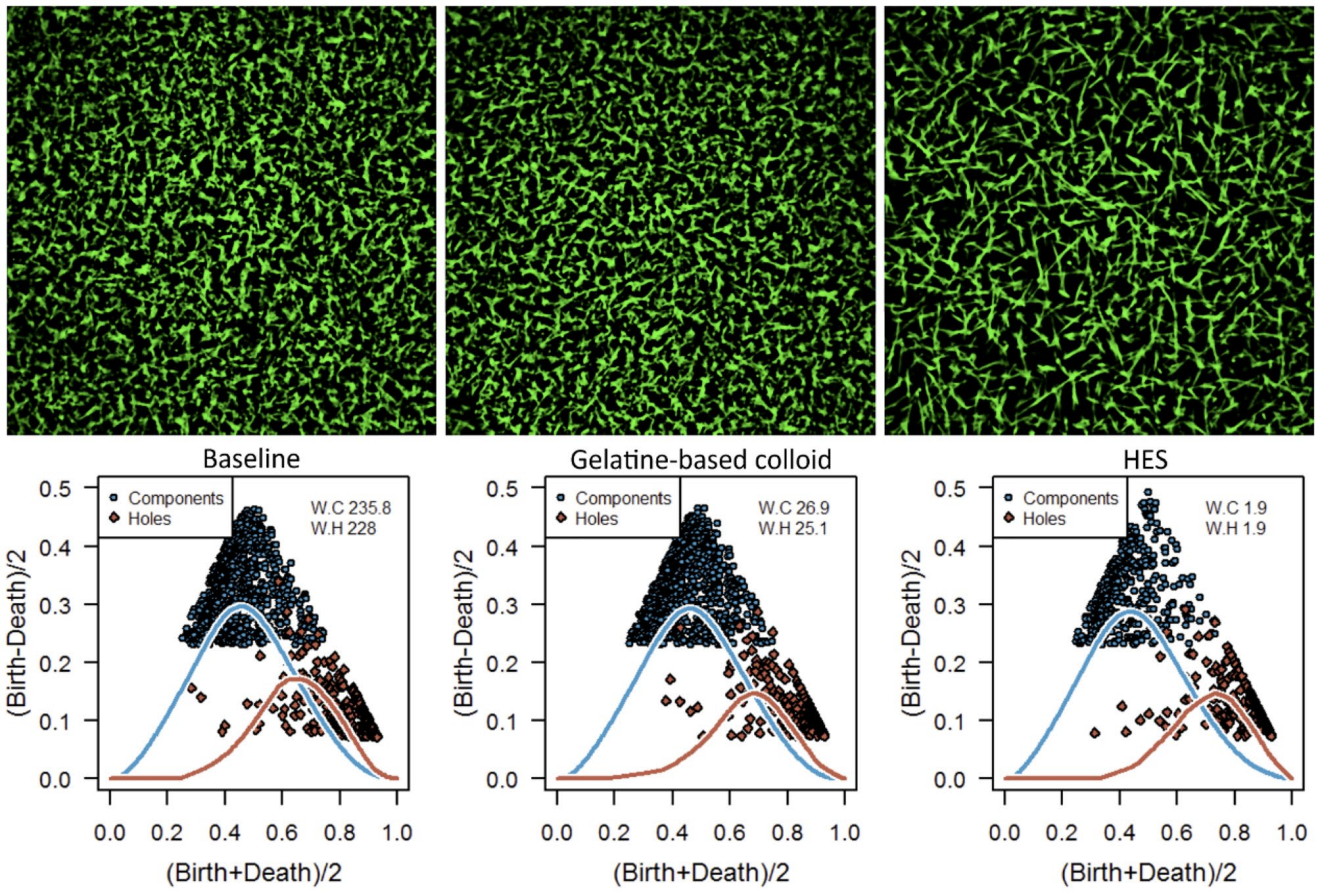
Porcine (pooled plasma) fibrin network without dilution (Baseline, left) and after 30% dilution with gelatine (centre) or hydroxyethyl starch (HES, right). Corresponding persistence diagrams are presented in the second row.

Figure 3 shows a porcine (pooled plasma) fibrin network without dilution (left), after 30% dilution with a gelatin-based colloid (center) or diluted with low-molecular weight HES (right), including the corresponding persistence diagrams. Microscopic visualization of the fibrin networks show minimal differences between baseline and the network diluted with colloids as they maintain their fine and dense structure. However, after dilution with HES, the network presents a more rigid structure. The respective persistence diagrams render this behavior, since the diagrams of the baseline and the gelatin-diluted samples are comparable, while the diagram of HES-diluted samples show fewer components. Evaluating the silhouettes, however, nearly no difference can be observed.

The fewer components due to dilution with HES were also reflected in viscoelastic tests since the reduction of maximum clot firmness (MCF) compared to baseline measurement (both p < 0.05) was higher with HES (BL 25 ± 2 vs. 4 ± 3 mm after dilution) than with the gelatin-based colloid (BL 27 ± 7 vs. 12 ± 4 mm after dilution).

For components, the Wasserstein distance from the empty diagram, i.e. the diagonal, verifies these observations that the baseline is comparable to gelatin, but not to HES (see Table 1). This is reflected by the long distance of components between baseline and HES. The differences in the Wasserstein distance for holes from the diagonal is less blatant, nonetheless the distance between baseline and HES is again higher than the distance between baseline and gelatin-based colloid. Looking at the fractal dimension, no distinction between baseline and gelatin, and a marginal difference between baseline and HES is observed. Explicitly counting the number of components again shows similar results between baseline and gelatin-based colloid while the network after dilution with HES has less than half the number of components. The number of holes, however, is comparable for all three networks.

**Table 1:** Parameters of components analysis at baseline and after dilution of porcine plasma.

	Baseline	Gelatine-based colloid	HES	Baseline – gelatine-based colloid	Baseline – HES
Wasserstein distance^a^					
Components	235.76	227.98	122.10	13.64	115.53
Holes	26.93	25.10	28.19	4.66	7.52
Fractal dimension	1.86	1.86	1.84	0.00	0.03
Number of components	629	612	288	17	341
Number of holes	206	190	194	16	12

^a^Wasserstein distance from diagonal for Baseline and after dilution with either gelatine-based colloid or HES;

### Argatroban induced topological differences

**Figure 4:**
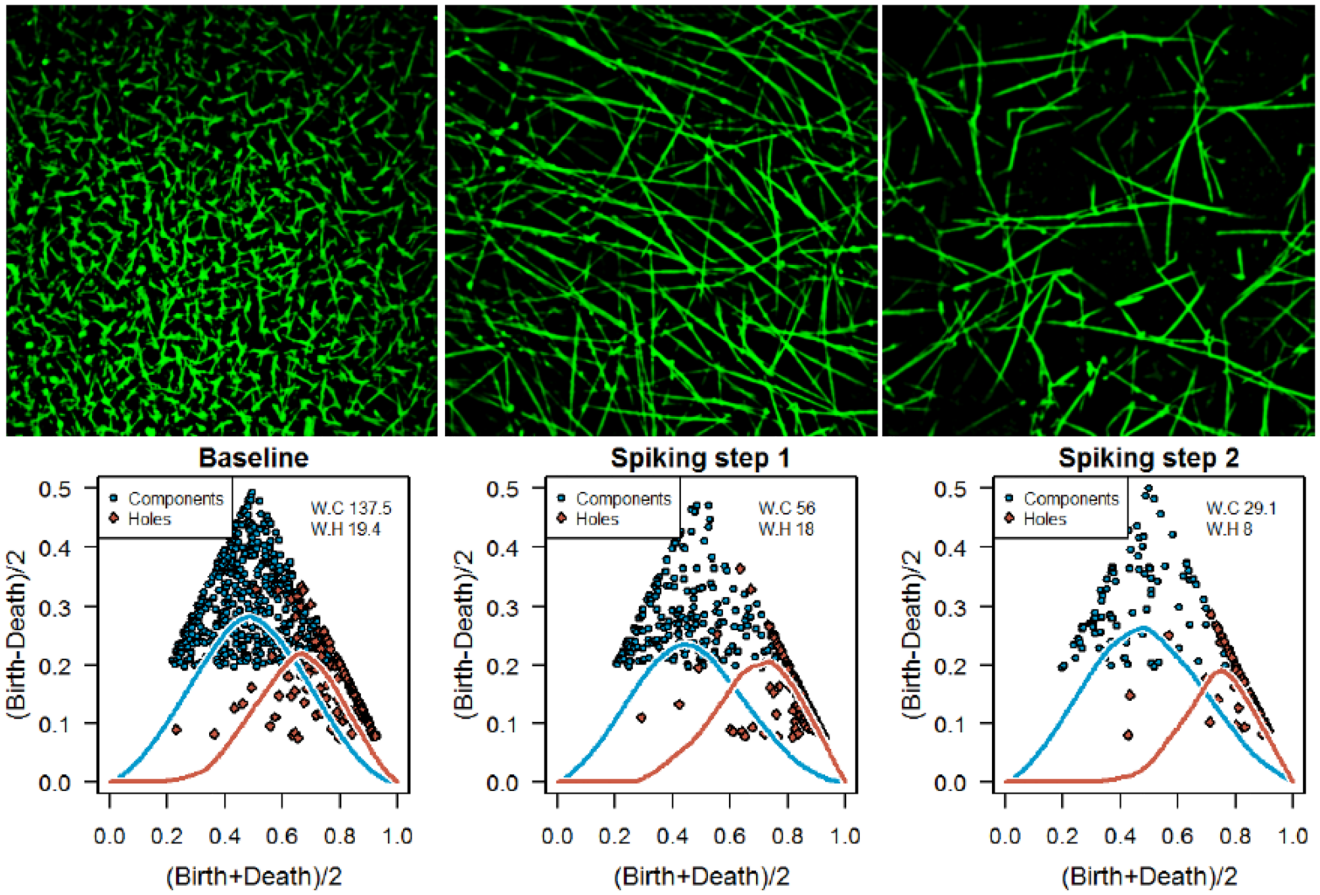
Baseline human fibrin network (green, left) compared to networks imaged after addition of 0.5 µg/mL (spiking step 1) and 1.0 µg/mL argatroban (spiking step 2). Corresponding persistence diagrams are presented in the second row, where components are represented by blue squares, holes by red diamonds and the lines represent the associated silhouettes.

Figure 4 shows the fibrin network at baseline (left) and networks after spiking step 1 with 0.5 μg/mL (center) and spiking step 2 with 1.0 μg/mL (right) argatroban, as well as the corresponding persistence diagrams. The structure of the networks impose a loss of complexity after each spiking step, while the thickness of the filaments increases. This subjective observation is reflected by TDA, as the persistence diagrams indicate a decrease in distinct features with increasing doses of argatroban.

The observed differences between the networks were verified by the measures presented in Table 2. The Wasserstein distances reveal significant differences in the components for both spiking steps and a significant difference from baseline to the second spiking step in the holes. The mean Wasserstein distance from the baseline persistence diagram compared to spiking step one (S1) and spiking step two (S2) is 59.44 (41.61 to 77.26) and 71.24 (47.73 to 94.75) for components and 9.69 (7.36 to 12.02) and 9.5 (7.49 to 11.51) for holes, respectively. This is in accordance with the pairwise differences. The fractal dimensions show only small differences among the natural network, the first spiking step (S1), as well as in the second spiking step (S2). While a significant difference between baseline and S1 is observed, no significant alterations are detected between baseline and S2. Counting components of the binarized images shows a decrease in the number of components after spiking, which is not statistically significant. Counting the number of holes captures significant differences between baseline and S1 and baseline and S2. Due to the more rigid structure after the first spiking step, more holes emerge, while the second spiking step reduces the number of fiber cross points of the network and hence decreases the number of holes again.

**Table 2:** Wasserstein distances from diagonal, pairwise Wasserstein distance, fractal dimension, number of components and holes.

	Baseline	S1	S2	Baseline – S1		Baseline – S2	
				Estimate^a^	p value^b^	Estimate^a^	p value^b^
Components	144.82 (120.21 to 169.42)	109.21 (87.53 to 130.88)	101.91 (81.61 to 122.22	35.61 (8.70 to 62.52)	0.012	42.90 (9.03 to 76.78)	0.016
Holes	20.81 (16.62 to 25.01)	17.39 (13.82 to 20.95)	17.03 (13.17 to 20.89)	3.43 (-0.62 to 7.47)	0.093	3.78 (0.14 to 7.43)	0.042
Fractal dimension	1.82 (1.81 to 1.84)	1.81 (1.80 to 1.83)	1.81 (1.80 to 1.83)	0.01 (0.00 to 0.03)	0.038	0.01 (0.00 to 0.03)	0.094
Number of components	447.05 (277.12 to 616.97)	370.41 (275.76 to 465.06)	374.73 (329.80 to419.66)	76.64 (-113.99 to 267.27)	0.413	72.32 (-104.91 to 249.54)	0.406
Number of holes	158.64 (109.96 to 207.31)	187.00 (92.14 to 281.86)	110.64 (83.82 to 137.45)	-28.36 (-140.60 to 83.87)	0.014	48 (-12 to 108)	0.001

S1 = Spiking 1, S2 = Spiking 2; ^a^Mean pairwise difference with corresponding 95% CI. ^b^Assessed by paired t-test.

## Discussion

This study shows for the first time that the mathematical method of topological data analysis is suitable to assess differences in the architecture of fibrin networks due to pharmacological treatments.

TDA is a computational tool able to describe multivariate interactions, a feature that many traditional methods, such as principal component analysis or network analysis lack. The topological formation of a fibrin network is dependent on an array of factors, including pH, temperature, viscosity, osmolality and of course, all zymogens and cofactors of the coagulation cascade, which are known for their many ways of interaction^26,27^. The formation and crosslinking of the fibrin strands is therefore a product of multidimensional interactions, a characteristic that hinders to predict the shape and the physical as well as the physiological features of the resulting network.

Here, we applied two clear modifications: by adding argatroban to the samples, the functional activity of only one isolated factor was inhibited: thrombin. Thrombin is a key enzyme in the coagulation cascade. Its most prominent function is the conversion of fibrinogen to fibrin, but it also catalyzes several positive and negative feedback loops within the coagulation cascade, influencing both velocity of clot formation while also inducing self-limiting mechanisms and even the activation of fibrinolytic pathways, which work towards the breakdown of the fibrin network^28^. The second modification was dilution of the samples. The activity of all participating factors to clot formation is unaltered, only the concentration is affected. There is a single causal modification in both experimental set ups, but due to the nature of the coagulation system, the effect is multidimensional.

The successful mathematical description of fibrin images was first shown by Pretorius et al., who clustered fibrin fibers according to their thickness and could show a difference between different species^29^ and who also investigated the visual inspection of clot characteristics in different pathophysiologies^30^. The topological analysis of fibrin networks as alternative method was shown by Berry E et al. who was able to topologically describe differences of fibrin networks of different species^31^.

The structural changes after dilution of pooled porcine plasma as well as the topological variations induced by argatroban are captured in the persistence diagrams.

Visual assessment conferred a notable difference to argatroban treated samples. While the fibrin network imposed thinner, this therapeutic agent was shown to have a pro-fibrinolytic effect^32,33^ and this effect is attributed to an altered clot structure^33^. Notably, thrombin directly alters the clot structure and influences the resistance to fibrinolysis^34^. Our analysis revealed similar effects, since the branching points and therefore the components and holes of Wasserstein distances were decreasing with increasing thrombin inhibition. Lower thrombin generation is observed in patients with hemophilia and addition of coagulation FVIIa, which enhances the earlier phases of thrombin generation, were shown to alter the resulting fibrin network, with thinner, but more abundant connections^35^. The significance of early thrombin concentrations was computationally interpreted by analyzing microstructural templating mechanisms, also with means of fractal analysis but without applying persistent homology explorations to quantify and compare the differences^36^. Interestingly, initially lower thrombin levels resulted in incipient fibrin gels with a lower level of branching points of the fibrin network, which was not corrected during the propagation phase of the polymerization process. This analysis also highlighted that random fractal aggregates influenced the microstructure of the clot^36^. We speculate that probably also platelet function could be represented with this analysis technique, since platelets would cause random fractal aggregates. The dilution of porcine plasma samples with a gelatine-based colloid resulted in less obvious qualitative differences of the formed fibrin network, i.e. visually comprehensible reduction of branching points or fiber thickness, despite remarkable differences in viscoelastic assessments. Analysis by TDA in contrast could indeed show that dilution with gelatine or HES results in a significant increase in holes and voids and decreases the number of components in a clot. Thus, TDA of fibrin networks has the potential to reflect clot characteristics which are not described by the traditional methods of coagulation analysis.

We could show the feasibility of this mathematical approach to capture differences of the functionality of the coagulation system within a species by extracting information from multidimensional greyscale images. By adding a method to evaluate morphological characteristics of the end product of the coagulation process, we expect that this approach might provide a new avenue to explore pathophysiological subtypes in patients with coagulation dysfunctions. The TDA method might complement already available tools to investigate the functional properties of the coagulation system and, for instance, could be a helpful tool to evaluate the influence of various drugs on coagulation or to characterize the response to a certain treatment.

This is a small pilot study with the goal to establish a mathematical model to describe the fibrin network. Notably, there are significant differences regarding the coagulation system between pigs and humans^37,38^. Here, the same reagents were used for assaying human as well as porcine blood. For the in-vitro tests only blood of healthy donors was used. An additional limitation is that we concentrated on the analysis of a statically formed plasma clot. Flow and shear stress greatly influence the physical properties of a blood or a plasma clot^39,40^. Both of these physiological forces are not represented by our evaluation of statically formed blood clots. While the inclusion of shear provides important aspects on e.g. clot stability and lysis dynamics, excluding an arbitrarily defined shear force omits a potential source of variance regarding physical test properties as both flow and shear forces are influenced by rheological characteristics of the included sample which can vary greatly in different human pathologies^41,42^. An advantage of observing statically formed clots therefore is a certain degree of standardizability. Another advantage regards the scalability of this technique: citrated plasma is needed to perform global coagulation assays. We could imagine that future detection mechanisms could shift from turbidimetric or absorption-related to optical methods, which would allow a comprehensive analysis not only on clotting times but also on individual clot structures.

In conclusion, TDA is a mathematical approach useful for quantification of morphological differences of fibrin clots. It remains to be investigated in further studies how this new method may be integrated into scientific and clinical routine and how evaluation of fibrin network density could become an additional tool for identifying patients at risk for bleeding or thrombosis or treatment response in various diseases.

## Data Availability

Relevant data will be freely available to any researcher wishing to use them for non-commercial purposes, without breaching participant confidentiality. The datasets generated during and/or analysed during the current study are available from the corresponding author on reasonable request.
